# Nucleotide Modifications Decrease Innate Immune Response Induced by Synthetic Analogs of snRNAs and snoRNAs

**DOI:** 10.3390/genes9110531

**Published:** 2018-11-02

**Authors:** Grigory Stepanov, Evgenii Zhuravlev, Victoria Shender, Anna Nushtaeva, Evgenia Balakhonova, Elena Mozhaeva, Marat Kasakin, Vladimir Koval, Alexander Lomzov, Marat Pavlyukov, Irina Malyants, Mikhail Zhorov, Tatyana Kabilova, Elena Chernolovskaya, Vadim Govorun, Elena Kuligina, Dmitry Semenov, Vladimir Richter

**Affiliations:** 1 Institute of Chemical Biology and Fundamental Medicine, Siberian Branch, Russian Academy of Sciences, 630090 Novosibirsk, Russia; evgenijur@gmail.com (E.Z.); nushtaeva.anna@gmail.com (A.N.); zendriya-87@mail.ru (E.B.); mozhaevaalena@gmail.com (E.M.); kassakinm@gmail.com (M.K.); koval@niboch.nsc.ru (V.K.); lomzov@niboch.nsc.ru (A.L.); zhorovlbnk@yandex.ru (M.Z.); kabilova@niboch.nsc.ru (T.K.); elena_ch@niboch.nsc.ru (E.C.); kuligina@niboch.nsc.ru (E.K.); semenov@niboch.nsc.ru (D.S.); richter@niboch.nsc.ru (V.R.); 2 Department of Natural Sciences, Novosibirsk State University, 630090 Novosibirsk, Russia; 3 Shemyakin-Ovchinnikov Institute of Bioorganic Chemistry of the Russian Academy of Sciences, 117997 Moscow, Russia; victoria.shender@gmail.com (V.S.); marat.pav@mail.ru (M.P.); 4 Federal Research and Clinical Center of Physical-Chemical Medicine of Federal Medical-Biological Agency, 119435 Moscow, Russia; iricam@mail.ru (I.M.); vgovorun@yandex.ru (V.G.); 5 Faculty of Technology of Organic Substances and Chemical Pharmaceutical Compounds, Mendeleyev University of Chemical Technology of Russia, 125047 Moscow, Russia

**Keywords:** synthetic RNA analogs, small nuclear RNAs, small nucleolar RNAs, RNA modification, innate immune response, RNA-Seq, PKR

## Abstract

Short nuclear regulatory RNAs play a key role in the main stages of maturation of the precursors of the major RNA species. Small nuclear RNAs (snRNAs) form the core of the spliceosome and are responsible for the splicing of pre-mRNA molecules. Small nucleolar RNAs (snoRNAs) direct post-transcriptional modification of pre-rRNAs. A promising strategy for the development of non-coding RNA (ncRNAs) mimicking molecules is the introduction of modified nucleotides, which are normally present in natural ncRNAs, into the structure of synthetic RNAs. We have created a set of snoRNAs and snRNA analogs and studied the effect of base modifications, specifically, pseudouridine (Ψ) and 5-methylcytidine (m^5^C), on the immune-stimulating and cytotoxic properties of these RNAs. Here, we performed a whole-transcriptome study of the influence of synthetic snoRNA analogs with various modifications on gene expression in human cells. Moreover, we confirmed the role of PKR in the recognition of snoRNA and snRNA analogs using the short hairpin RNA (shRNA) technique. We believe that the data obtained will contribute to the understanding of the role of nucleotide modification in ncRNA functions, and can be useful for creating the agents for gene regulation based on the structure of natural snoRNAs and snRNAs.

## 1. Introduction

There are two major classes of short non-coding RNAs (ncRNAs) in eukaryotic cells: small nuclear RNAs (snRNAs), which form the core of spliceosomes and catalyze the removal of introns during pre-mRNA processing, and small nucleolar RNAs (snoRNAs), which, being a part of small nucleolar ribonucleoproteins (snoRNPs), direct post-transcriptional modifications of rRNAs [1,2,3,4,5]. Box C/D snoRNAs guide 2′-*O*-methylation, while box H/ACA snoRNAs are responsible for pseudouridylation of rRNA nucleotides that form functional active centers of the ribosome [6,7,8,9].

**Figure 1:**
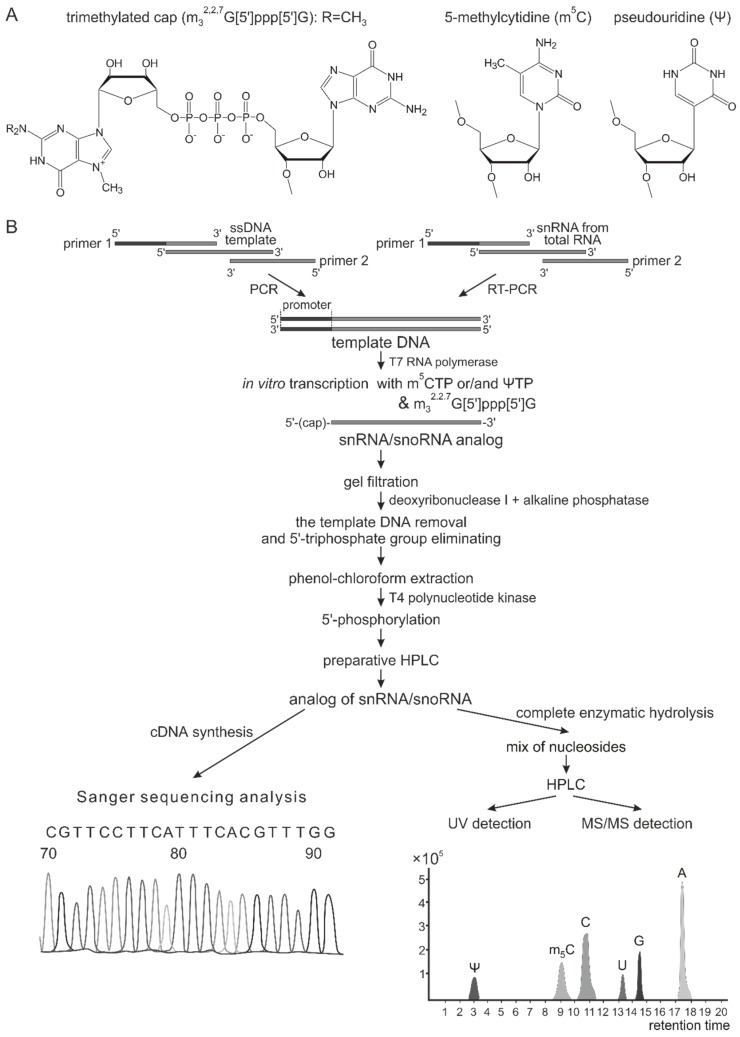
The scheme of synthesis of modified non-coding RNA analogs. (**A**) The structures of modified RNA nucleotides: Trimethylated cap analog (m_3_^2.2.7^G[5′]ppp[5′]G), 5-methylcytidine (m^5^C), and pseudouridine (Ψ). (**B**) The scheme of synthesis of artificial analogs of human snoRNAs and snRNAs.

All types of ncRNAs undergo post-transcriptional maturation, including selective chemical modification of nucleotides (Figure 1A). The significance of the modifications in the control of RNA functions and metabolism still has not been unambiguously established. However, recent studies have revealed the role of modified RNA monomers in RNA stability, localization, RNA-RNA, and RNA-protein interactions, as well as in the regulation of the activity of pattern recognition system components [10,11,12,13].

One of the approaches commonly used to study the properties and functions of ncRNAs is the in vitro synthesis of their analogs and subsequent transfection into human or animal cells. The key challenge in this strategy is non-specific activation of the cellular innate immune response system (interferon response) [14]. A cascade of interferon-dependent transcription activation events in the cells results in altered expression of a large number of protein-coding and non-coding genes, making it difficult to interpret the results of the experiments aimed to elucidate the direct effect of synthetic RNAs [15,16,17,18]. Therefore, the development of approaches to decrease the non-specific immune-stimulating activity of ncRNA analogs remains an important issue.

The study by Kariko et al. demonstrated that nuclear fraction of RNA isolated from mammalian cells induces lower innate immune response rate in monocyte-derived dendritic cells (DCs) than cytoplasmic and total cellular RNA fractions. At the same time, transfection of mitochondrial RNA into human cells resulted in a strong immune response [19]. On the one hand, these results suggest that nucleotide modifications play a key role in recognition of exogenous and native RNA molecules. On the other hand, they point out the rationale for using the structure of nuclear RNAs to create artificial gene expression modulators while nucleotide modification can be utilized to regulate its immune-stimulating activity. Several examples of the use of nuclear and nucleolar RNA structures for the construction of gene-specific regulators were demonstrated earlier [20,21,22,23,24,25,26].

Cytosolic accumulation of snoRNAs after stress stimuli has been recently reported. It was shown that a number of canonical snoRNAs could be released into the cytoplasm upon treatment with fatty acids and doxorubicin [27,28]. It is known that the presence of short single-stranded nuclear RNA species can be detected in the cytoplasm by various receptors, including PKR and RIG-I. One can assume that active transport of short nuclear RNA species can be one of the mechanisms of the lethal cellular immune response. Such a mechanism may involve intracellular RNA receptors and activation of RNase L, which, in turn, can provide amplification of the immune signal. The fragments of short RNA (<200 nt) produced by RNase L have been recently shown to activate interferon beta (IFN-β) expression and amplify antiviral innate immunity [29]. Such fraction of the short total RNA should apparently contain snoRNAs and snRNAs. Interestingly, some representatives of the snoRNA and snRNA species and their fragments are often found in the RNA fractions circulating in human body fluids both in physiological and pathological conditions [30,31,32,33,34]. Distributed throughout the organism with the flow of body fluids, these RNA molecules could trigger signal cascades within the recipient cells by interacting with cell-surface or intracellular receptors.

Effective suppression of immune-stimulating activity by nucleotide modifications was shown for mRNA analogs, siRNAs, and fragments of viral RNAs [35,36,37,38,39]. Earlier, we used Illumina microarray technology to study changes in gene expression in human breast adenocarcinoma cells (MCF-7) transfected with unmodified snoRNA analogs. We found that artificial box C/D snoRNAs induced a strong innate immune response. In particular, they upregulated transcription of the genes involved in the cellular response to viral infection and foreign genetic material, such as *RIG-I* (*DDX58*), *OAS1*, *MYD88*, *RNASEL*, *PKR* (*EIF2AK2*), interferon-induced proteins with tetratricopeptide repeats (*IFITs)*, as well as interferon-dependent transcription factor, STAT, and interferon-regulatory factor (IRF) families [40]. We proposed that innate immune activation may lead to the cytotoxic effect of box C/D snoRNA analogs, and found some features providing differences in the sensitivity of human cells to the action [41]. In addition, snoRNAs were shown to activate PKR in vitro and in vivo, indicating that these RNAs have a role in the regulation of the mechanisms of the immune response in eukaryotes [42]. Earlier, the data of TLR3 activation by ultraviolet-induced fragments of U1 snRNA have been presented [43].

In this study, we performed a whole-transcriptome analysis of gene expression in cells transfected with U25 snoRNA analogs, containing either only canonical (“non-modified”, “NM”) or modified (m^5^C instead of C and/or Ψ instead of U) nucleotides. Furthermore, we obtained similarly modified analogs of U35a snoRNA and U12 snRNA and demonstrated that modified nucleotides attenuated both immune-stimulating activity and cytotoxicity of exogenous RNAs. Having analyzed the relation between the observed effect and the level of modification, we noticed that Ψ to U substitution influences the stability of RNA structures that could provide preconditions for modeling the impaired interaction between RNA and cell-surface and intracellular receptors. Moreover, we confirmed the role of PKR in the recognition of both unmodified and partially modified snoRNA and snRNA analogs using human cells stably expressing shRNA targeted at mRNAs of pattern recognition receptors. Our results may have important implications for improving the design of RNAs that are capable of evading innate immune responses.

## 2. Materials and Methods

### 2.1. In Vitro Synthesis of Artificial RNAs

DNA templates for snoRNA analogs were synthesized via PCR with BioMaster HS-Taq PCR (Biolabmix, Novosibirsk, Russia) using an oligonucleotide template and primers, with 5′-primers containing the T7 RNA polymerase promoter sequence (see Supplementary Materials Primers). The templates for U12 snRNA analogs were obtained via reverse transcription followed by PCR (RT-PCR) of total RNA from MCF-7 cells with specific primers (see Supplementary Materials Primers) using one-step BioMaster RT-PCR-Color (Biolabmix). The amplification products were analyzed by electrophoresis in 2.5% agarose gel, and DNA sequences were verified with Sanger sequencing (the analysis of termination products was performed by SB RAS Genomics Core Facility, ICBFM SB RAS, Novosibirsk, Russia).

Artificial RNAs were synthesized by in vitro transcription with T7 RNA polymerase (Biosan, Novosibirsk, Russia) in the reaction mixture containing 2.5 ng/μL of purified PCR product, 40 mM Tris-HCl (pH 7.9), 6 mM MgCl_2_, 2 mM spermidine, 10 mM DTT, 2 mM NaCl, 0.40 U/μL of RiboLock RNase Inhibitor (Thermo Fisher Scientific, Waltham, MA, USA), 1.0 mM each of canonical NTPs (Biosan) or ΨTP (instead UTP) or m^5^CTP (instead CTP), and 2 mM m_3_^2.2.7^G[5′]ppp[5′]G trimethylated cap analog (Jena Bioscience, Jena, Germany) (last only for capped analogs). To obtain RNAs with partial substitution of a modified nucleotide for its canonical equivalent, the transcription reaction was performed in a reaction mix in which the ratio of one particular modified NTP relative to the corresponding unmodified NTP was 20% (m^5^CTP), 30% (ΨTP), or 50% (both m^5^CTP and ΨTP).

In vitro transcription products were purified from low molecular weight components using Illustra MicroSpin G-25 columns (GE Healthcare, Chicago, IL, USA). The synthetic RNA solutions were treated with DNase I (Thermo Fisher Scientific) and alkaline phosphatase (Thermo Fisher Scientific) to remove DNA templates and 5′-triphosphate moieties. The ncRNA analogs were isolated with phenol/chloroform extraction using Lira Reagent (Biolabmix). To obtain snoRNA and snRNA analogs with 5′-monophosphate (for non-capped analogs), artificial RNAs were treated with the 20 U T4 polynucleotide kinase (Biosan) in the buffer containing 0.1 mM ATP, 10 mM DTT, 2.5 mM MgCl_2_, 10 mM Tris-HCl (pH 7.5), and 0.1 mM CaCl_2_. Finally, RNA transcripts were purified via ion-pair reversed-phase high-performance liquid chromatography (IPRP HPLC, 0.15 mL/min flow rate, 40 °C, linear gradient from 100% A to 100% B in 10 min: Eluent A—0.05 M aqueous solution of triethyl ammonium acetate (TEAA) (pH 7.5); eluent B — 0.05 M solution of TEAA in a 20% solution of acetonitrile (pH 7.5)) on a Milichrome A-02 liquid chromatograph (Econova, Novosibirsk, Russia) with a multi-wavelength UV-detector using a 2.0 × 7.5 mm column with the ProntoSIL-120-5-C18 sorbent (Econova) and precipitated by ethanol with 0.6 M NaOAc. RNA concentrations were determined spectrophotometrically on Nanodrop (Thermo Fisher Scientific) and verified using “Qubit” fluorometer with an RNA BR Assay Kit (Thermo Fisher Scientific). RNA integrity was verified on 2.5% agarose or 8% denaturing polyacrylamide gels using visualization by SYBR Green (Thermo Fisher Scientific) or 5′-[^32^P]-label (Laboratory of biotechnology ICBFM SB RAS, Novosibirsk, Russia). Synthetic RNAs were stored in diethylpyrocarbonate (DEPC)-treated water solution at −70 °C. RNA sequences were verified with reverse transcription and subsequent Sanger cDNA sequencing, with the product being analyzed by SB RAS Genomics Core Facility (ICBFM SB RAS). The presence and amount of modified monomers were verified by HPLC-MS/MS and HPLC-UV of nucleosides after enzymatic digestion of artificial RNAs (Figure 1).

### 2.2. Enzymatic Digestion of RNAs and HPLC-UV Analysis

Synthetic RNA analogs (1.0–5.0 µg) were digested with 0.65 mg/mL of ribonuclease A (Thermo Fisher Scientific), 4.0 µg/mL nucleases P1 (Sigma-Aldrich, St. Louis, MO, USA), 0.2 mg/mL phosphodiesterase I (Sigma-Aldrich) and 2 U alkaline phosphatase (Thermo Fisher Scientific) per 1 µg RNA in 10 mM Tris-HCl (pH 8.0), 5.0 mM MgCl2, 0.1 M KCl, and 0.02% Triton X-100, 0.1 mg/mL BSA at 37 °C for 5–20 h. The deactivation of enzymes was carried out by heating at 75 °C for 10 min and chloroform extraction. The aqueous phase containing nucleosides was collected and was dried in Eppendorf vacuum concentrator 5301 (Eppendorf, Hamburg, Germany). The pellet was dissolved in DEPC-treated water. Samples were analyzed with HPLC-UV (0.15 mL/min flow rate, 40 °C, eluent A—0.05 M aqueous solution of TEAA; eluent B—0.05 M solution of TEAA in a 20% acetonitrile: Linear gradient from 100% A to 100% B in 15 min) on a Milichrome A-02 liquid chromatograph (Econova) or HPLC-MS/MS (as described below). Control nucleosides were obtained from corresponding nucleoside triphosphates (Jena Bioscience) under the same conditions. Using HPLC-UV, it was found that synthetic RNAs obtained in the mixture with 20% m^5^CTP and 30% ΨTP contained 13–15% m^5^C and 25–28% Ψ; analogs obtained in the mixture with 50% m^5^CTP and 50% ΨTP contained 50–52% m^5^C and 52–54% Ψ.

### 2.3. HPLC-MS/MS

During HPLC-MS/MS, nucleosides were resolved on an Eksigent ekspert microLC 200 system (Eksigent Technologies, Dublin, CA, USA) with a Zorbax 300 SB-C18 (4.6 mm ID × 150 mm) column (Agilent Technologies, Santa Clara, CA, USA) by elution with the following gradient of acetonitrile in water: 0.2 mL/min flow rate, 40 °C, eluent A—water, eluent B—acetonitrile: 100% A for 2 min, 1–20% B for 2–6 min, 20–30% B for 6–8 min. The HPLC column was coupled to an AB Sciex 3200QTRAP mass spectrometer (AB SCIEX, Framingham, MA, USA) with an electrospray ionization source. The ions were detected in positive ion mode. The retention time, *m*/*z* of the transmitted parent ion, *m*/*z* of the monitored product ions for modified nucleosides was: Ψ, 1.7 min, *m*/*z* 245→125; m^5^C, 2.5 min, *m*/*z* 258→126. The identities of individual nucleosides were established by comparison with standards obtained (as described above) and using literature data [44,45]. The processing of the results of LC-MS/MS was performed by the software, Analyst 1.62 and MultiQuant 2.1 (AB SCIEX).

### 2.4. Transfection of Human Cells with Synthetic RNAs

Human breast adenocarcinoma cells, MCF-7, obtained from Russian Cell Culture Collection of Vertebrates (Institute of Cytology, Russian Academy of Sciences, St. Petersburg, Russia) were cultured in Iscove’s Modified Dulbecco’s Media (IMDM) (Invitrogen, Carlsbad, CA, USA) supplemented with 10% fetal bovine serum (FBS) (Biolot, St. Petersburg, Russia), 10 mM l-glutamine, and 40 μg/mL gentamicin (Sigma-Aldrich). RNA transfections were carried out with Lipofectamine RNAiMAX (Invitrogen) cationic lipid delivery vehicles. For Lipofectamine RNAiMAX transfections, the required amount of synthetic RNAs (a final concentration of 10 nM in medium) and 1.5 μL reagent per 1 mL of culture medium were first pooled and then diluted in 15 μL of PBS. This mixture was incubated for 15 min at room temperature before being dispensed to the culture medium. The cells were incubated at 37 °C in a humidified atmosphere with 5% CO_2_ for 24 h. At time points of 24 h, total RNA was isolated from the cells using Lira Reagent (Biolabmix) according to the manufacturer’s protocol. The accumulation of synthetic RNAs into cells was confirmed by RT-PCR using specific primers and BioMaster RT-PCR SYBR Blue (Biolabmix).

### 2.5. Differential Gene and Transcript Expression Analysis

Whole transcriptome analysis with sequencing was performed using polyA fraction of total cellular RNA on an Illumina HiSeq 1500 platform by Genoanalytica Company (Moscow, Russia). Sequencing data (fastq formatted reads) were applied to the “classic” RNA-Seq workflow, which includes read mapping with TopHat (hg19), and assembly with Cufflinks (RefSeq). The comparison of expression levels of genes and transcripts in RNA-Seq experiments was carried out with CuffDiff [46]. The list of differentially expressed genes (CuffDiff FDR adjusted after Benjamini-Hochberg correction of *p*-value for multiple-testing *q* < 0.05) were applied to the gene enrichment analysis powered by the Enrichr platform [47].

### 2.6. qRT-PCR

qRT-PCR reaction was performed with BioMaster RT-PCR SYBR Blue (Biolabmix, Novosibirsk, Russia) by LightCycler 96 System (Roche, Basel, Switzerland). The primers used in the study are presented in the Supplementary Materials. The levels of mRNAs were represented as relative values normalized to the level of GAPDH and HPRT mRNA. Quantitative PCR data analysis was done using qbase+ software, version 3.1 (Biogazelle, Zwijnaarde, Belgium), which includes finding the stable reference genes with geNORM, quality control, and relative quantification of mRNA levels of genes [48,49]. The mean values (±standard deviation (SD)) from 3 independent experiments were represented. The PCR products were analyzed on 1.5% agarose gel.

### 2.7. Thermal Denaturation Experiments

Thermal denaturation experiments of 1.0 μM concentration of RNA were carried out in PBS buffer (pH 7.0) using Cary 300 Bio (Varian, Australia) spectrophotometer. Heating and cooling of the samples were performed in the range 5–95 °C, with the temperature change rate of 1 °C/min. Absorbance at 260, 270, and 300 nm was measured. Absorbance at 300 nm was used as a baseline and was subtracted from the two other denaturation curves [50]. The melting temperature (Tm) of RNAs was calculated as the maxima of the first derivative of absorbance on temperature. The values obtained at 260 and 270 nm during heating and cooling were averaged.

### 2.8. MTT Assay

To estimate the influence of the synthetic RNAs on MCF-7 human adenocarcinoma cell viability and proliferation we used MTT assay. Aliquots (150 mL) containing 2.5 × 10^3^ cells were plated onto 96-well plates and incubated at 37 °C with 5% CO_2_. After 24 h, the cells were transfected with 10 nM artificial RNAs (complexed with Lipofectamine RNAiMAX). After incubation for 24 h, MTT (3-(4,5-dimethyl-2-thiazolyl)-2,5-diphenyl-2*H*-tetrazolium bromide) (Sigma-Aldrich) was added to the cells at a final concentration of 0.5 mg/mL and the plates were incubated at 37 °C for 3 h. The medium was removed, followed by the addition of 0.15 mL of DMSO to each well. The plates were read at 570 and 620 nm using an Apollo LB912 plate reader (Berthold Technologies, Oak Ridge, TN, USA). Cell viability was determined as the absorbance at 570 nm with reference to 620 nm and expressed as a percentage of control (control cells were incubated with Lipofectamine RNAiMAX only) ±SD for triplicate independent experiments.

### 2.9. iCELLigence Assay

The proliferation rate and survival of A549 human cells with shRNA-mediated PKR, RIG-I, and MDA5 knockdown as well as cells expressing scrambled shRNA (scr-shRNA), under snoRNA and snRNA analogs transfection were analyzed using the iCELLigence RTCA System (ACEA Bioscience, San Diego, CA, USA). Cells were plated in 8-well E-plates (ACEA Bioscience) at a density of 5000 cells per well in a total volume of 200 µL of IMDM, and were monitored in real-time mode (Figure S1). After the initial 24 h of growth, the culture medium was replaced with fresh IMDM with complexes of Lipofectamine RNAiMAX with snoRNA and snRNA analogs. The data was recorded every 15 min for 48 h post transfection, and cell indexes were calculated by RTCA Software 1.2 (ACEA Bioscience). Cell index is a parameter reflecting the impedance of electron flow caused by adherent cells. Relative cell indexes (RCI) for each ncRNA analogs were calculated as values relative to control incubated with Lipofectamine RNAiMAX only and normalized to effect on cells expressing scr-shRNA.

### 2.10. Statistical Analysis

The statistical significance of the differences in experiments was determined using the two-tailed Student’s *t*-test (data are presented as means ± SD). Differences were considered statistically significant at *p*-value < 0.05.

## 3. Results

### 3.1. Synthesis of Modified ncRNA Analogs

To analyze the effect of modified ncRNA on gene expression, we obtained synthetic analogs of human U25 and U35a snoRNAs as well as U12 snRNA using in vitro transcription. An approach previously used in [23] was improved for the incorporation of modified nucleotides and elimination of the 5′-terminal triphosphate in RNA-transcripts. Also, we excluded heating stages to avoid RNA degradation and managed to achieve a higher yield of artificial RNAs. The ncRNA analogs obtained differed from each other in the content of Ψ and m^5^C, as well as in the structure of the 5′-terminal nucleotide, namely, the synthetic RNAs bore 5′-OH, 5′-monophosphate, or trimethylated cap (Figure 1). RNA sequences were analyzed using reverse transcription and subsequent Sanger cDNA sequencing, while the presence of modified monomers was verified by HPLC-MS/MS analysis, with the quantitative evaluation performed by HPLC-UV detection of nucleosides after enzymatic digestion of in vitro transcripts [45].

### 3.2. RNA-Seq Analysis of Human MCF-7 Cells Transfected with Ψ- and m^5^C-Modified RNAs

We used ncRNA analogs for transfection of MCF-7 human breast adenocarcinoma cells and analyzed changes in gene expression. To determine the effect of synthetic ncRNAs with modified nucleotides on the innate immune response in human cells, we first performed Illumina RNA-Seq gene expression analysis of MCF-7 cells transfected with non-capped (with 5′-OH) U25 snoRNA analogs without modifications and analogs modified by complete substitution of Ψ for U or m^5^C for C.

**Figure 2:**
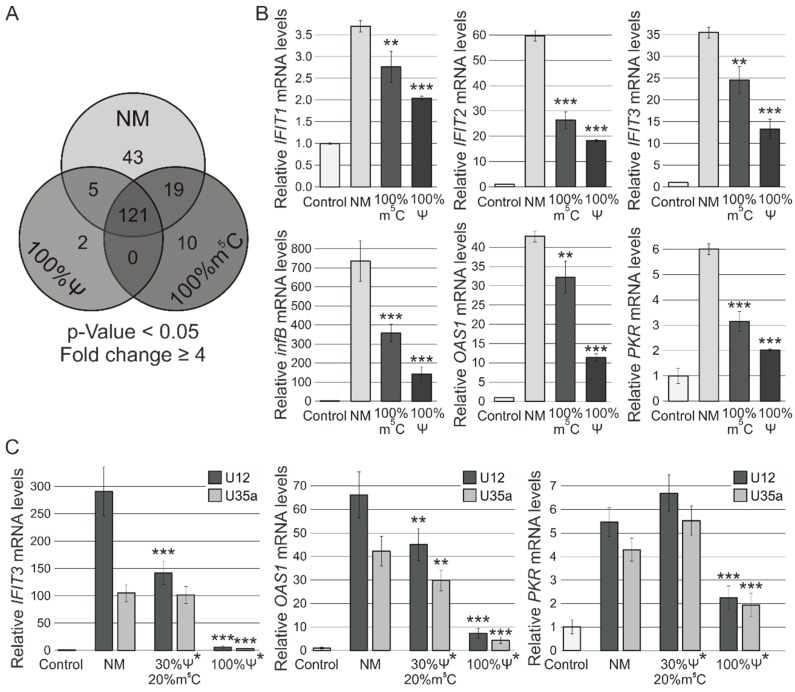
Summary and validation of RNA-Seq data. (**A**) The Venn diagrams showing the number of statistical significant (*p*-value < 0.05) changes in gene expression after MCF-7 cells transfection with a synthetic non-modified U25 box C/D snoRNA (**NM**) and analogs containing Ψ instead of U (**100%Ψ**) or m^5^C instead of C (**100%m^5^C**). The genes are listed in Supplementary Table S1. (**B**) qRT-PCR data showing relative expression levels of interferon-regulated *IFIT1, IFIT2, IFIT3, infB, OAS1,* and *PKR* in MCF-7 cells 24 h after transfection with a non-modified (**NM**) and two modified (**100%Ψ** and **100%m^5^C**) analogs of U25 box C/D snoRNA. (**C**) qRT-PCR data showing relative expression level of *IFIT3, OAS1,* and *PKR* mRNA in MCF-7 cells 24 h after transfection with non-modified (**NM**) and m^5^C- and Ψ-containing (**20% m^5^C/30%** **Ψ** and **100%Ψ**) analogs of human U12 snRNA and U35a snoRNA. Control cells were incubated with Lipofectamine RNAiMAX only. The error bars represent standard deviations. The asterisks (*) indicate analogs with additional trimethylated m_3_^2,2,7^G caps. The difference between the **NM** and **20% m^5^C/30%** **Ψ** or **100%Ψ** groups was statistically significant at *p*-value < 0.05 (**) and at *p*-value < 0.01 (***).

Differential expression analysis of RNA-Seq data revealed that transfection with the non-modified U25 snoRNA analog resulted in changes in the expression of 188 genes (*p*-value < 0.05, Fold change ≥ 4) in MCF-7 cells (Figure 2). Modified snoRNA analogs containing Ψ and m^5^C caused changes in expression of 128 and 150 genes (*p*-value < 0.05, Fold change ≥ 4), respectively. As demonstrated in Figure 2A, a total of 121 genes were common for all three types of snoRNAs. Functional enrichment analysis revealed that this group of genes was predominantly involved in such processes as signaling pathways of type I and II interferons and negative regulation by host of viral infection (including GO:0060337, GO:0045071, GO:0045869, GO:0044828, GO:0060333, GO:0032480, GO:0071346, GO:0035458, and GO:0034341, with *p*-value ≤ 5.0 × 10^−10^, see Supplementary Table S1). Using Enrichr service [47], we demonstrated that observed gene expression changes were regulated by interferon-dependent IRF9 (*p*-value = 3.93 × 10^−4^; adj. *p*-value = 3.62 × 10^−2^) and IRF3 (*p*-value = 1.71 × 10^−8^; adj. *p*-value = 1.61 × 10^−6^) transcription factors. Interestingly, the group containing 43 genes specific for the non-modified synthetic RNA transfection was shown to be regulated by two factors mentioned above and, in addition, by NF-κB1 (*p*-value = 1.87 × 10^−6^; adj. *p*-value = 4.96 × 10^−4^) and HDAC2 (*p*-value = 3.50 × 10^−4^; adj. *p*-value = 1.71 × 10^−2^), suggesting the possibility that the epigenetic mechanisms of gene regulation were involved after transfection. Moreover, the sets of genes activated by each of analogs contained predicted targets of miR-146a-5p (adj. *p*-value ≤ 8.22 × 10^−12^), which is known as a negative regulator of innate immune activation (see Table S1).

### 3.3. RT-PCR Verification of Gene Expression Changes

Together with a decrease in the total number of activated genes, a diminution of the individual activation level of the key genes involved in the innate immune response was observed after using modified U25 RNA analogs. The differences in the gene expression were confirmed by qRT-PCR (Figure 2B, Figure S2). The most significant increase in the expression was shown for *infB* while m^5^C and Ψ modifications reduced its up-regulation by two- and five-folds, respectively. Other analyzed genes, *IFIT1*, *IFIT2*, *IFIT3*, *OAS1*, and *PKR*, also demonstrated lower mRNA levels in the cells transfected with modified U25 RNA compared to a non-modified analog. Thus, the expression of *IFIT2* was found to decrease from 60-fold (NM) to 27-fold (m^5^C) and 18-fold (Ψ); the expression of *IFIT3* decreased from 35-fold (NM) to 25-fold (m^5^C) and 13-fold (Ψ). Even though the modified analogs caused a similar effect, it is evident that complete substitution of Ψ for U resulted in the most significant effect on suppression of the cellular innate immune response.

In order to confirm our evidence on the ability of m^5^C and Ψ modifications to decrease the immune-stimulating activity of synthetic ncRNAs, we obtained modified analogs of human U12 snRNA and U35a snoRNA with additional trimethylated m_3_^2,2,7^G cap similar to those in native ncRNAs [51]. Along with ncRNA containing a complete substitution of U to Ψ, we also obtained U12 and U35a analogs with a 30% and 20% content of Ψ and m^5^C, respectively. We showed that the differences in *IFIT3* and *OAS1* activation observed between the intact and modified RNAs were more significant in the case of these U35a and U12 analogs, which were longer than U25 RNA analogs (Figure 2C).

### 3.4. Analysis of Cytotoxicity and Antiproliferative Effects of Synthetic Modified ncRNAs

**Figure 3:**
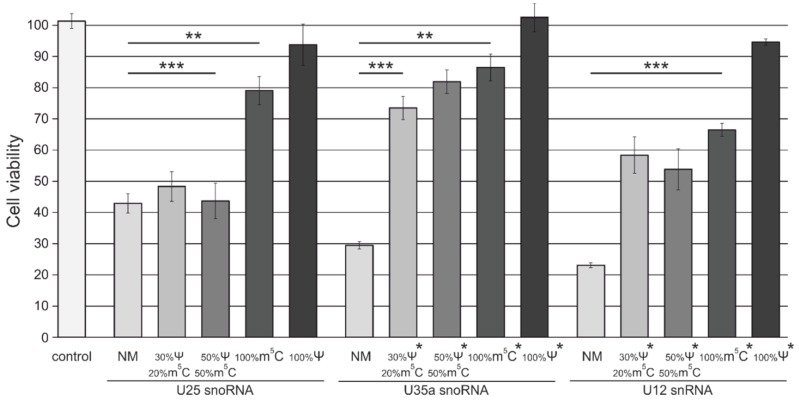
The viability of MCF-7 cells 24 h after transfection with snRNA and snoRNA analogs. Cells were transfected with 10 nM of non-modified (**NM**) and m^5^C- and Ψ-containing (**20% m^5^C/30%** **Ψ**, **50% m^5^C/50% Ψ**, **100% m^5^C** and **100%** **Ψ**) U25, U35a snoRNAs, and U12 snRNA analogs with Lipofectamine RNAiMAX. The asterisks (*) indicate analogs with additional trimethylated m_3_^2,2,7^G cap. Control cells were incubated with Lipofectamine RNAiMAX only. Data are presented as the mean of at least three independent experiments. The error bars represent standard deviations. The difference between the “control” and transfected cell groups was statistically significant at *p* < 0.05 (**) and at *p* < 0.01 (***).

We next analyzed how the reduction in immune-stimulating activity of synthetic ncRNAs affected the cytotoxicity of these RNAs. We transfected MCF-7 cells with snoRNA and snRNA analogs, and performed an MTT-test 48 h after transfection. It was found that transfection with unmodified ncRNA analogs decreased the viability of MCF-7 cells up to 42%, 30%, and 22% in the case of U25, U35a snoRNA analogs, and U12 snRNA analog, respectively. According to their cytotoxicity observed, synthetic ncRNAs were aligned in a row: “NM” > “30% Ψ/20% m^5^C” ≈ “50% m^5^C/50% Ψ” ≥ “100% m^5^C” > “100% Ψ”. As shown in Figure 3, complete substitution of U with Ψ diminished the cytotoxic effect caused by snoRNA and snRNA analogs 48 h after transfection.

Earlier, we obtained A549 human cells with shRNA-mediated *PKR (PKRkd)*, *RIG-I*, and *MDA5* knockdown (Figure S3). In this work, the lines were used for determining the role of the pattern recognition receptors in providing cytotoxic activity of artificial snoRNA and snRNA analogs. Initially, we showed that MCF-7 and A549 cells are similar in the profile of activation of innate immune gene expression (Figure S4). Using xCelligence technology, we analyzed antiproliferative effects of U35a and U12 analogs on the A549 cells, stably expressing shRNAs targeted at *PKR*, *RIG-I*, and *MDA5*, and *scr-shRNA*. The same cell lines incubated with Lipofectamine RNAiMAX only were used as the control.

**Figure 4:**
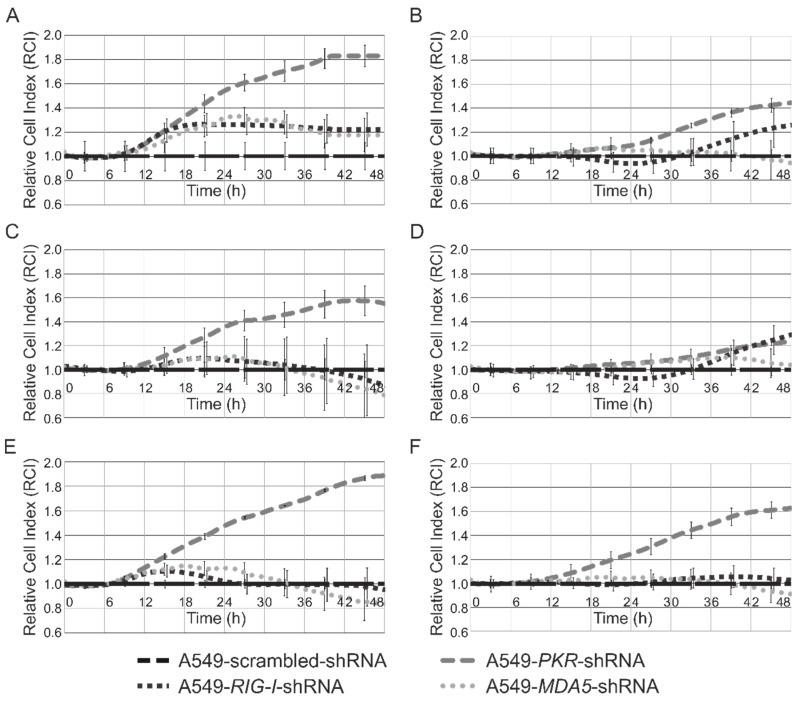
Curves showing the cell index of A549 human cells stably expressing one of shRNAs targeted at *PKR*, *RIG-I*, and *MDA5*, relative to that of cells expressing scrambled shRNA upon transfection with U35a and U12 analogs: Non-modified U35a (**A**), Ψ-modified U35a without cap (**B**), non-modified U35a with cap (**C**), Ψ-modified of U35a with cap (**D**), non-modified U12 (**E**), and Ψ-modified U12 (**F**). Relative cell indexes (RCI) are presented as the average means, with the error bars representing standard deviations.

It was found that knockdown of *PKR* resulted in the greatest effect on the differences of cell lines transfected with unmodified U35a and U12 analogs (Figure 4A,E, respectively). Capping of U35a analog (Figure 4C,D compared to Figure 4A,B, respectively) and modification of U35a and U12 analogs with Ψ resulted in a substantial reduction in differences between *PKRkd* and control cells expressing *scr-shRNA*. (Figure 4B,D,F compared to Figure 4A,C,E, respectively). Suppression of *MDA5* and *RIG-I* genes was not found to significantly change the sensitivity of A549 cells. Changes in the sensitivity of human cells to synthetic RNA were also indicated by the time period of the appearance of differences between the control line with *scr-shRNA* and the lines with knockdown. So, noticeable deviations in the growth curves (RCI > 1.2) were observed after 16 h of transfection with unmodified analogs, although modification with Ψ delayed the same effect to 30 and 24 h for U35a and U12 analogs, respectively.

### 3.5. Thermal Denaturation Studies

**Figure 5:**
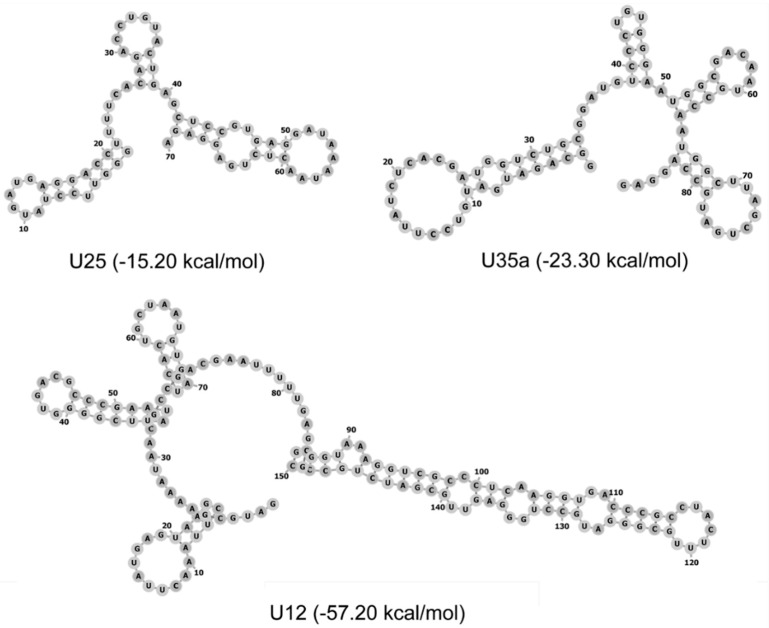
Predicted secondary structure of U25, U35a snoRNAs, and U12 RNA using the Vienna RNA package 2.0 [52]. In the brackets, the values of the Gibbs free energy at 37 °C are shown.

**Figure 6:**
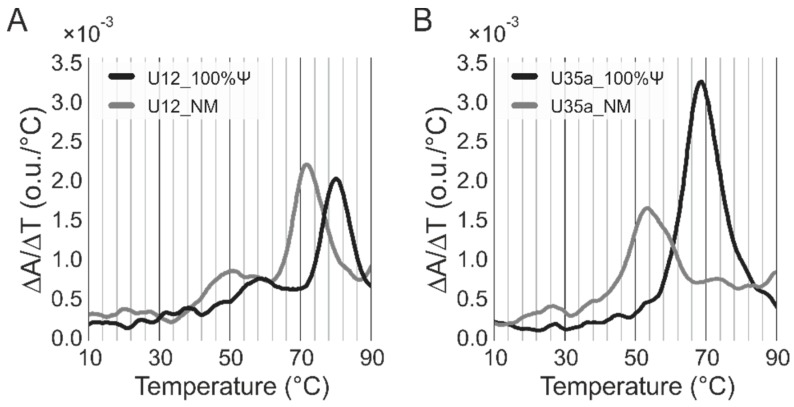
Differential thermal denaturation curves obtained during heating experiments at 260 nm of non-modified (**NM**) and Ψ-containing (**100%** **Ψ**) U12 snRNA (**A**) and U35a snoRNA (**B**) analogs.

To clarify the structural effects caused by modified nucleotides, we studied the thermodynamic stability of U35a snoRNA and U12 snRNA analogs. First, using the ViennaRNA Package 2.0 [52], we simulated spatial structures of the RNA analogs containing only canonical nucleotides (Figure 5). However, we did not detect any common structural features that could explain the effect of incorporating modified nucleotides in RNAs on the immune response activation and cytotoxicity. Therefore, we further determined the thermodynamic stability of the secondary structures of ncRNAs in solution using thermal denaturation studies. We demonstrated that the complete substitution of Ψ for U resulted in significant stabilization of the RNA secondary structure. We observed the significant increase in the Tm from 53.5 to 69.0 °C for the U35a analog, and from 71.5 to 80.0 °C for the U12 RNA analog (Figure 6). Therefore, we supposed that it is these observed structural thermodynamic properties of modified RNA analogs that could play the key role in the interaction between synthetic RNAs and RNA-binding proteins, such as extra- and intracellular receptors. A similar effect of the Ψ on the thermodynamic stability of RNA molecules was described earlier [53,54,55].

## 4. Discussion

Synthetic RNAs are known to activate the pathways of the innate immune response upon transfection into human cells [14]. Activation of the expression of interferon-sensitive genes in mammalian cells by RNA analogs is provided by a set of RNA-binding receptors, including TLRs, RIG-I, MDA-5, and PKR, with MDA-5 and TLR3 recognizing dsRNAs [13,14,56,57]. The 5′-triphosphate moiety significantly enhances an activation stimulus of RNAs due to its ability to interact with RIG-I and PKR receptors, as well as IFIT antiviral proteins [58,59,60,61,62]. To eliminate undesirable side effects induced by 5′-triphosphate, we used the scheme of synthesis of ncRNA analogs, allowing us to remove or substitute it with 5′-monophosphate or cap (Figure 1).

We observed activation of the innate immune response in human cells upon transfection with non-modified and Ψ- or m^5^C-modified U25 snoRNA analogs. As expected, the strongest effect was caused by the analog without modifications, while m^5^C and Ψ substitutions led to a decrease in the interferon-dependent cellular response. We found that observed gene expression changes induced by each of three types of U25 RNA analogs were regulated by interferon-dependent IRF9 and IRF3 transcription factors. Specifically, the group of 43 genes, which were upregulated in the cells transfected with the unmodified U25 analog, included an additional transcription factor, NF-kB. Analysis of known activation mechanisms involved in immune-stimulating pathways allowed us to propose that modifications mainly affected RNA interaction with RIG-I and PKR. To better understand the mechanisms of suppression of the innate immune response, we synthesized m_3_^2,2,7^G-capped, Ψ-, and/or m^5^C-modified analogs of other short ncRNAs, namely, U35a snoRNA and U12 snRNA. U35a was selected from the list of the box C/D snoRNAs that mediated the metabolic stress response after entering the cytoplasm induced by saturated free fatty acids. We assumed the possibility of transferring the function of this snoRNA not only to the cytoplasm, but also outside the cell [28]. U12 snRNA was detected in ovarian cancer ascites, suggesting it may have an additional extracellular regulatory role [32]. It was found that the presence of nucleotide modifications in U35a and U12 analogs also resulted in decreased immune-stimulating and cytotoxic activity. The selected modifications are known to be capable of inhibiting the signal transmission through PKR and RIG-I-like receptors [37,63]. At the same time, RNA-dependent activation of TLR7/8 has been shown to be significantly inhibited by m^5^C/Ψ-modifications, while TLR3 is still activated by a modified RNA. Considering our results of thermal denaturation experiments, we can speculate that the main role of RNA modifications in decreasing the immune response is connected with the significant increase in the thermodynamic stability of secondary structure elements of synthetic RNAs and, as a consequence, impaired rearrangements in the RNA-receptor complex that are necessary for the activation of signal transduction through the adapter proteins. In support of this hypothesis, a recent study has demonstrated that Ψ modification inhibits binding of minimally structured RNA to specific proteins due to the reduced RNA flexibility [55]. Using the shRNA technique and xCelligence method, we confirmed the role of *PKR* in the recognition of snoRNA and snRNA analogs. We found that the suppression of *PKR* expression significantly reduced the cytotoxic effect of unmodified RNA. Modification of ncRNA analogs, especially the simultaneous complete substitution of Ψ for U and the capping, allowed minimization of the antiproliferative effect and the difference between control cells and the *PKRkd* line, showing that these modifications were able to inhibit PKR-dependent pathways. Downregulation of *MDA5* and *RIG-I* genes was not found to significantly change the sensitivity of human cells. Our data are in good agreement with general concepts that RIG-I and MDA5 recognize RNAs with 5′-triphosphate and dsRNAs, respectively, and with a recent work describing the interaction of some snoRNAs with PKR [13,42,59]. Our results showed that PKR can be activated not only by unmodified snoRNAs and snRNAs, but also by modified RNAs without cap, with nucleotide modifications reducing and slowing down the cytotoxic and antiproliferative effects of such RNAs.

On the one hand, our data might reveal the role of minor nucleotides in native small ncRNAs. On the other hand, the results can be used in the development of artificial regulators of gene expression based on the structure of short regulatory RNAs. Recent data indicated that RNA modifications could serve as regulators that tune the intracellular transport and guide the metabolism of the nuclear forms of short ncRNAs, for example, the release of these RNAs into the cytoplasm without activation of the innate immune response. Interestingly, Ψ sites have been found in many snoRNAs with the help of high-throughput sequencing [64,65,66]. By analogy with snRNA, one can assume that such modifications are necessary for the assembling of the proper secondary structure of short RNAs and fine-tuning of the interaction between an snoRNA and a target RNA in the nucleus and, perhaps, cytoplasm [5,51]. It has also been shown that ncRNAs could acquire modifications that are more typical for mRNA, such as m5С and m6A [67,68]. According to the results of the transcriptome-wide mapping of these modifications, the total pool of snRNAs and snoRNAs contains dozens of m5С and m6A sites. In the case of snoRNAs, more than 25% of both classes of box C/D and box H/ACA snoRNAs were found to contain at least one m6A [68]. The m6A modification is more common for mRNA, and this modification may have a possible role in RNA transport from the nucleus into the cytoplasm and transmission of the information about the subsequent metabolism of the RNA molecule. A recent study has shown that co-transcriptional mRNA m6A modification can regulate the rate of the translation and transmit such regulatory information from the nucleus into the cytoplasm [69]. Another study has shown that m6A interferes with the 15.5k-induced folding of box C/D k-turns into snoRNA molecules [70]. One can assume that modifications of the snoRNA nucleotides can alter its interaction network, leading to the impaired assembly of canonical snoRNPs and thus provide interaction of ncRNAs with novel protein partners [71], and even alter their localization within the cell. In this case, an snoRNA is not capable of forming a functional snoRNP, and it might probably be transported from the nucleus to perform other, still unknown, functions in the cytoplasm and/or be subjected to degradation and/or be exported from the cells within extracellular vesicles. Another possibility is that snoRNA modifications can be formed during transcription of the host gene and depend on the transcription rate. In this case, firstly, they can lead to the formation of the short-lived ncRNA-forms, which degrade alongside with the lasso structure of the excised intron of the host gene. Secondly, further disruption of the snoRNP assembly may serve as an additional link between central processes for the transfer of genetic information. In addition to the interaction between transcription in the nucleus and translation rate in the cytoplasm described by Slobodin and coauthors [69], snoRNA modifications can remotely connect the processes occurring in the nucleus with the processes of ribosome maturation, which take place in the nucleolus. It is of interest that other ways of acquiring the signs of mRNA by ncRNA species have been described in their metabolism regulation, for example, polyadenylation of ncRNA, which precedes the stages of directed degradation of nuclear RNA species [72,73,74].

Regarding the gene therapeutic relevance, it should be noted that activation of the innate immune response is usually considered as an undesirable side effect. However, immune-stimulating properties, as well as off-target mechanisms of si/shRNAs, may also have therapeutic implications and can be used in combination therapy [41,75,76,77,78]. We must understand the mechanisms of the functioning of synthetic ncRNA analogs to use them within the cell. As for the snoRNAs, it has been recently shown that PKR can play an important role in activation of the innate immune response through snoRNA analogs [42]. It is of interest that the presence of a 5′-triphosphate was shown to enhance PKR activity for several, but not all, analyzed snoRNAs. PKR activation in cells upon snoRNA transfection supported the hypothesis that endogenous snoRNAs could also activate PKR. We have found that base modifications reduce the immune-stimulating activity of the snoRNA and snRNA analogs containing a 5′-monophosphate or 5′-cap (Figure 2) as well as the 5′-triphosphate moiety (data not shown). Our data are in good agreement with the previously published results that certain modifications of synthetic RNAs introduced in vitro and found naturally abrogate activation of RNA-dependent innate immune factors. Small nucle(ol)ar RNAs are promising models for RNA-therapeutic development since they have a potency to fine-tune the processes of post-transcriptional RNA maturation and thereby regulate gene expression in a cell-recipient.

## 5. Conclusions

We have demonstrated that snoRNA and snRNA analogs activate a significant innate immune response upon transfection into human cells, while incorporation of Ψ and m^5^C nucleotides provided a reduction in the level of non-specific immune activation. Moreover, we showed that PKR can be activated not only by unmodified snoRNAs and snRNAs, but also by modified RNAs without a cap. Our data can be useful in the development of therapeutically relevant RNA agents for gene regulation based on the structure of snoRNA and snRNA species.

## Supplementary Materials

The following are available online at http://www.mdpi.com/2073-4425/9/11/531/s1. Table S1: Results of functional enrichment analysis. Figure S1: Growth curves of A549 human cells with shRNA-mediated PKR. Figure S2: RT-PCR data showing the level of *HPRT, GAPDH, IFIT3, TLR3* mRNA in MCF-7 cells 24 h after transfection with non-modified (**NM**) and Ψ- or m^5^C- containing (**100%** **Ψ**, **100% m^5^C**) analogs of U25 snoRNA. Figure S3: Inhibition of the expression of PRRs by shRNA in transduced A549 cell lines. Figure S4: qRT-PCR data showing relative expression level of some innate immune response genes in MCF-7 and A549 cells 24 h after transfection with non-modified analog of human U25 snoRNA. The primers used in the study are presented in Supplementary Materials Primers.
