# Supplementary material for: Nucleotide Modifications Decrease Innate Immune Response Induced by Synthetic Analogs of snRNAs and snoRNAs
Source: Genes (Basel). 2018 Nov 2;9(11):531. doi: 10.3390/genes9110531 (PMC6266926; doi:10.3390/genes9110531)
Supplement: Supplementary file 1 [file genes-09-00531-s001.zip › genes-381208 Supp Final/Supplementary/Supplementary Figure 1.docx]

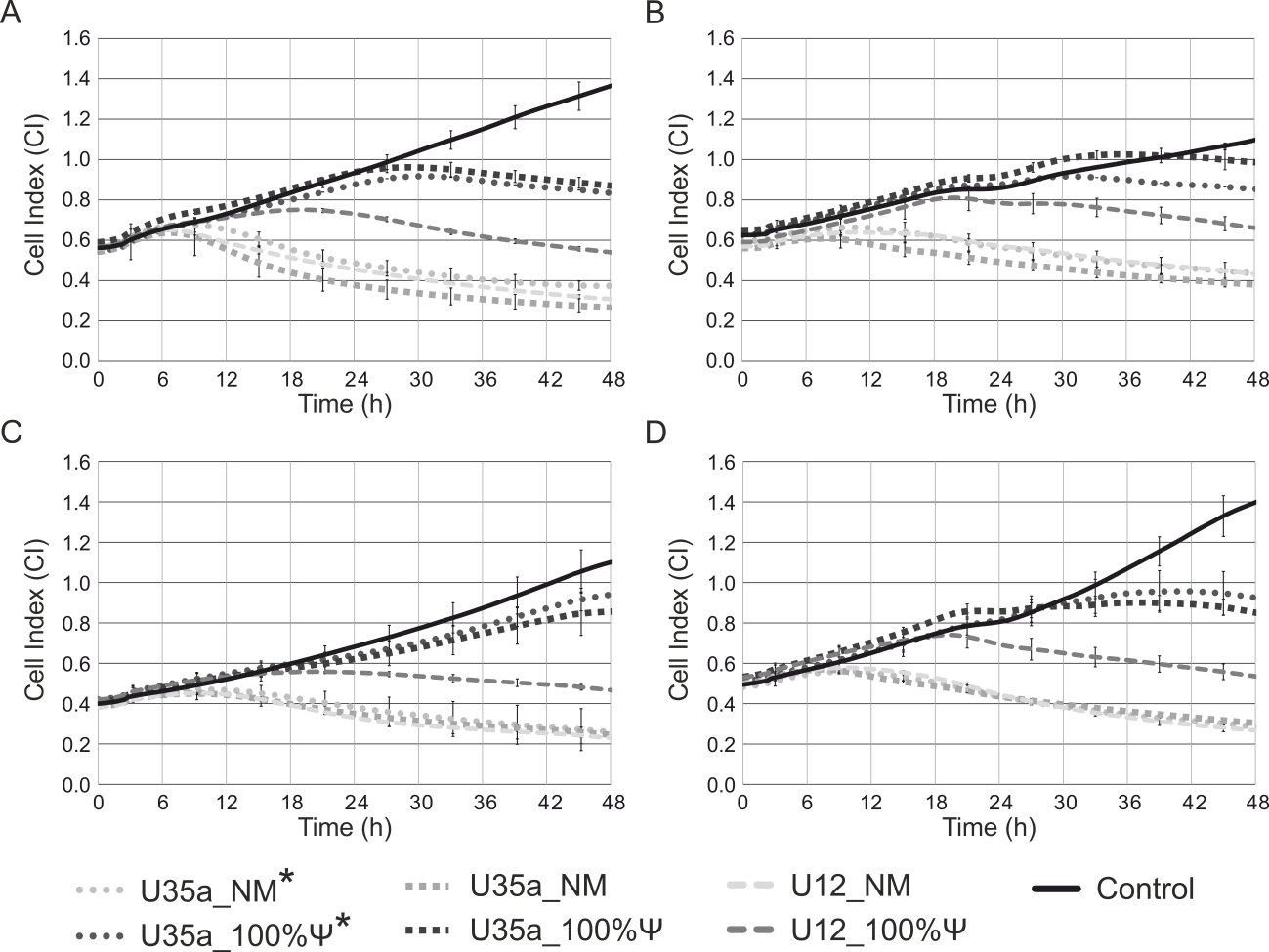


**Supplementary Figure 1.** Growth curves of A549 human cells with shRNA-mediated PKR (**B**), RIG-I (**C**) and MDA5 (**D**) knockdown, as well as control cells expressing scrambled shRNA (**A**) upon transfection with snoRNA and snRNA analogs. Cell indexes are presented as the average means, with the error bars representing standard deviations. Control cells were incubated with Lipofectamine RNAiMAX only. The asterisks (*) indicate analogs with trimethylated m_3_^2,2,7^G caps.
