# Supplementary material for: Nucleotide Modifications Decrease Innate Immune Response Induced by Synthetic Analogs of snRNAs and snoRNAs
Source: Genes (Basel). 2018 Nov 2;9(11):531. doi: 10.3390/genes9110531 (PMC6266926; doi:10.3390/genes9110531)
Supplement: Supplementary file 1 [file genes-09-00531-s001.zip › genes-381208 Supp Final/Supplementary/Supplementary Figure 2.docx]

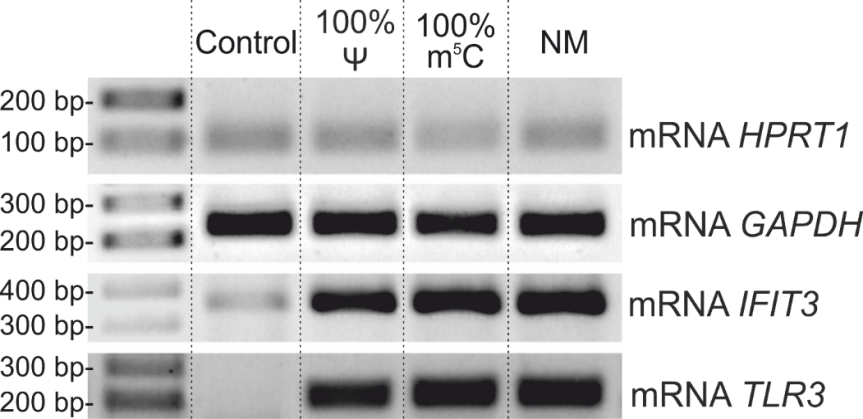


**Supplementary Figure 2.** RT-PCR data showing the level of *HPRT, GAPDH, IFIT3, TLR3* mRNA in MCF-7 cells 24 h after transfection with non-modified (**NM**) and Ψ- or m^5^C- containing (**100% Ψ**, **100% m^5^C**) analogs of U25 snoRNA. Control cells were incubated with Lipofectamine RNAiMAX only. RT-PCR products were analyzed in a 1.5% agarose gel.
