# Supplementary material for: Nucleotide Modifications Decrease Innate Immune Response Induced by Synthetic Analogs of snRNAs and snoRNAs
Source: Genes (Basel). 2018 Nov 2;9(11):531. doi: 10.3390/genes9110531 (PMC6266926; doi:10.3390/genes9110531)
Supplement: Supplementary file 1 [file genes-09-00531-s001.zip › genes-381208 Supp Final/Supplementary/Supplementary Figure 3.docx]

*Western blot assay.* Cells were lysed in buffer: 50 mM Tris (pH 8.0), 5 mM EDTA, 150 mM NaCl containing 0.1% SDS, 1x complete protease inhibitor cocktail (Roshe Diagnostics, Rotkreuz, Switzerland) and 1 mM PMSF (Sigma-Aldrich, St. Louis, MO, USA). Total protein extracts were analyzed by 10% SDS-PAGE gel and transferred to a Trance-Blot nitrocellulose membrane (Bio-Rad Laboratories, Hercules, CA, USA) by a wet blotting procedure (150 V, 400 mA, 60 min, 15 °C) using the ‘Mighty small transphor’ (GE Healthcare, Chicago, IL, USA). The membranes were incubated with primary antibodies at 4 °C overnight using the following concentration: PKR (2.8 µg/mL, ab32052, Abcam, Cambridge, UK), RIG-I (10 µg/mL, ab45428, Abcam) or MDA5 (2.9 µg/mL, ab126630, Abcam) followed by HRP-conjugated secondary goat anti-rabbit antibodies (5 µg/mL, AF5718, R&D Systems, Minneapolis, MN, USA) using iBind Western System (Thermo Fisher Scientific, Waltham, MA, USA). Visualization of bound antibodies was achieved by means of Novex ECL Chemiluminescent Substrate Reagent Kit (Thermo Fisher Scientific) using the C-DiGit Blot Scanner (LI-COR Biosciences, Lincoln, NE, USA).


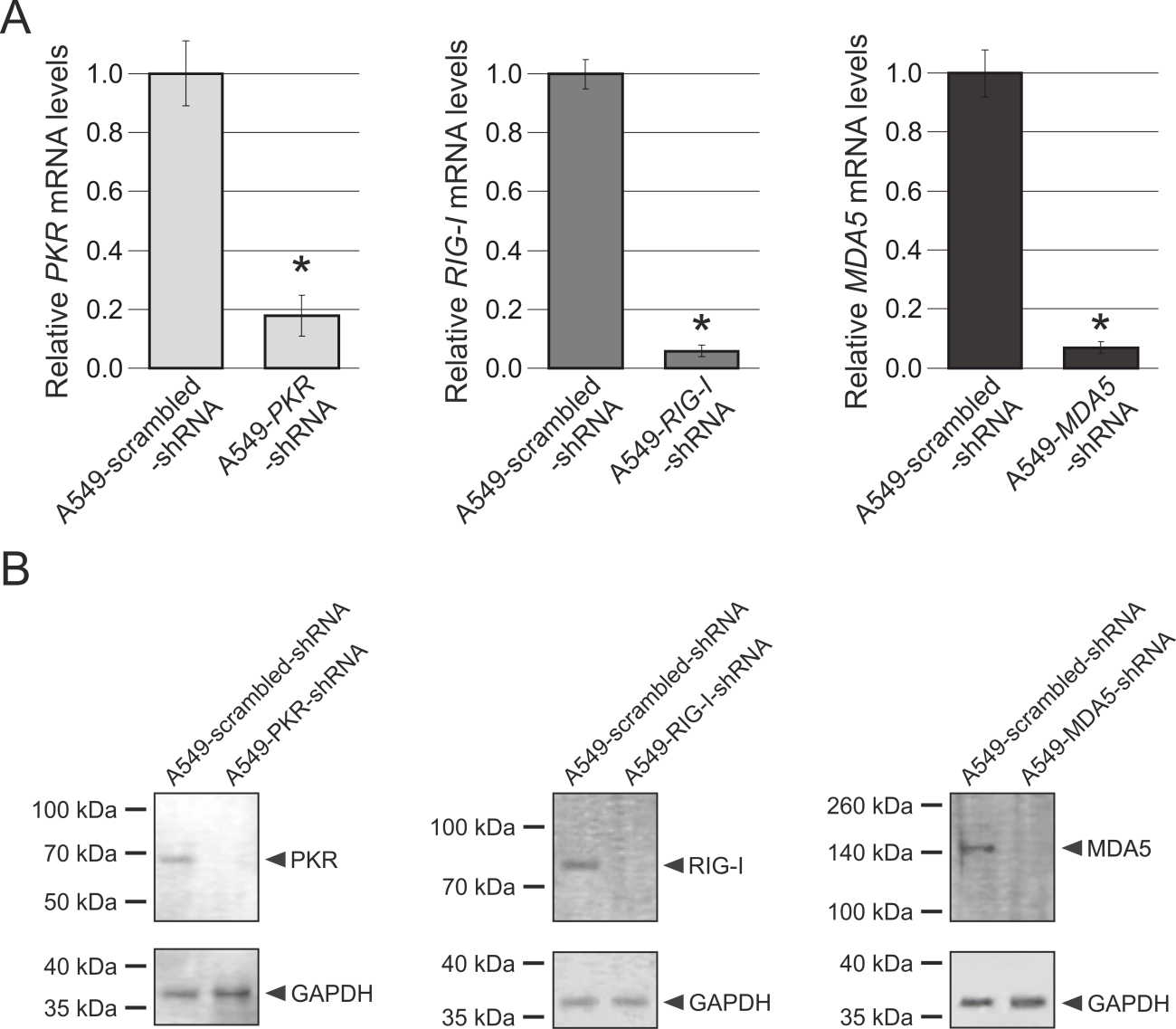


**Supplementary Figure 3.** Inhibition of the expression of PRRs by shRNA in transduced A549 cell lines. (A) qRT-PCR data showing relative expression level of mRNA of target genes in scrambled-shRNA-expressing A549 cell sublines. The error bars represent standard deviations. The difference between the A549-scrambled-shRNA and A549-target-shRNA was statistically significant at p-Value <0.01 (*). (B) Western blot analysis showing PKR, RIG-I, MDA5 and GAPDH expressions in A549 human cells with shRNA-mediated PKR, RIG-I and MDA5 knockdown, as well as control cells expressing scrambled shRNA. Cells were analyzed under innate immune activation condition via 24 h after transfection with a non-modified analog of human U25 snoRNA.
