# Supplementary material for: Nucleotide Modifications Decrease Innate Immune Response Induced by Synthetic Analogs of snRNAs and snoRNAs
Source: Genes (Basel). 2018 Nov 2;9(11):531. doi: 10.3390/genes9110531 (PMC6266926; doi:10.3390/genes9110531)
Supplement: Supplementary file 1 [file genes-09-00531-s001.zip › genes-381208 Supp Final/Supplementary/Supplementary Figure 4.docx]

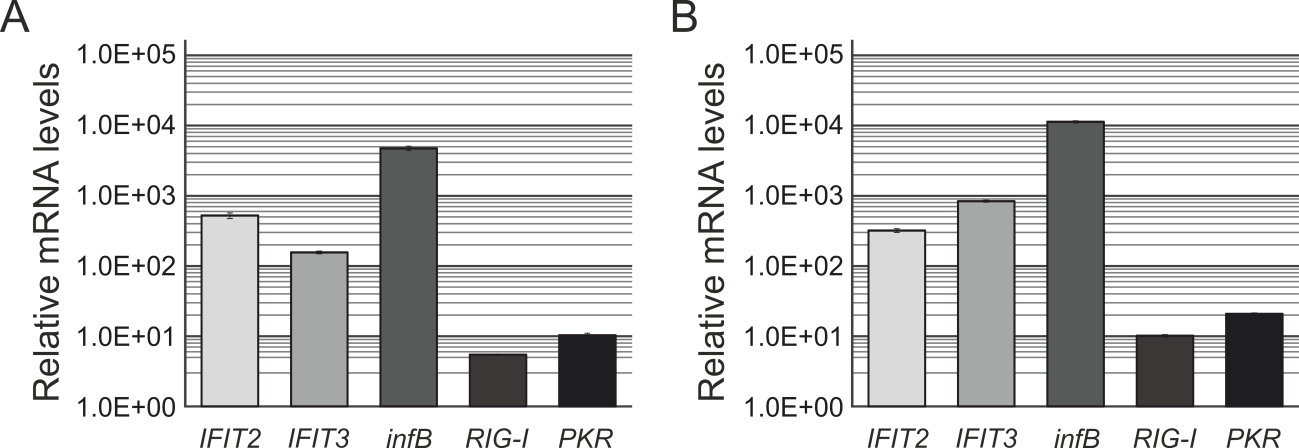


**Supplementary Figure 4.** qRT-PCR data showing relative expression level of some innate immune response genes in MCF-7 (**A**) and A549 (**B**) cells 24 h after transfection with non-modified analog of human U25 snoRNA. Control cells were incubated with Lipofectamine RNAiMAX only. The error bars represent standard deviations.
