# Supplementary material for: Nucleotide Modifications Decrease Innate Immune Response Induced by Synthetic Analogs of snRNAs and snoRNAs
Source: Genes (Basel). 2018 Nov 2;9(11):531. doi: 10.3390/genes9110531 (PMC6266926; doi:10.3390/genes9110531)
Supplement: Supplementary file 1 [file genes-09-00531-s001.zip › genes-381208 Supp Final/Supplementary/Supplementary Materials Primers.docx]

***Oligonucleotide primers for RT-PCR***

**Gene *GAPDH*:**

GAPDH-1 – 5ʹ-GAAGATGGTGATGGGATTTC-3ʹ

GAPDH-2 – 5ʹ-GAAGGTGAAGGTCGGAGT-3

**Gene *IFIT3*:**

IFIT3_1.1 – 5ʹ-GGCAGACAGGAAGACTTCTG-3ʹ

IFIT3_1.2 – 5ʹ-TTTCTGCTTGGTCAGCATGT-3ʹ

**Gene *OAS1*:**

OAS1_F – 5ʹ-CGATCCCAGGAGGTATCAGA- 3ʹ

OAS1_R – 5ʹ-TCCAGTCCTCTTCTGCCTGT-3ʹ

**Gene *PKR*:**

PKR_F – 5ʹ-TCGCTGGTATCACTCGTCTG- 3ʹ

PKR_R – 5ʹ-GATTCTGAAGACCGCCAGAG- 3ʹ

**Gene *IFIT1*:**

IFIT1_F – 5ʹ-AAAAGCCCACATTTGAGGTG-3ʹ

IFIT1_R – 5ʹ-GAAATTCCTGAAACCGACCA-3ʹ

**Gene *IFN-β*:**

IFNB_F – 5ʹ-CATTACCTGAAGGCCAAGGA-3ʹ

IFNB_R – 5ʹ-CAGCATCTGCTGGTTGAAGA-3ʹ

**Gene *HPRT1*:**

HPRT_1 – 5ʹ-CATCAAAGCACTGAATAGAAAT-3ʹ

HPRT_2 – 5ʹ-TATCTTCCACAATCAAGACATT-3ʹ

**Gene *IFIT2*:**

IFIT2_1.1 – 5ʹ-CATTTATTGGTGGCAGAAGA-3ʹ

IFIT2_1.2 – 5ʹ-GCTCTTGCTGGATTAACTCT-3ʹ

***Oligonucleotide template and primers for DNA templates synthesis by PCR***

***SNORD25***

**U25_base**5ʹ-TTCCTATGATGAGGACCTTTTCACAGACCTGTACTGAGCTCCGTGAGGATA-3ʹ

**U25_T7 –** 5ʹ-ATGCAGCTAATACGACTCACTATAGGGTTCCTATGATGAGGACCT-3ʹ

**U25_R –** 5ʹ-TCTCCTCAGAGTTATTTATCCTCACGGAGCTCAG-3ʹ

***SNORD35a***

**U35a_base**5ʹ-CAGATGATGTCCTTATCTCACGATGGTCTGCGGATGTCCCTGTGGGAATGGCGA-3ʹ

**U35a_T7 –** 5ʹ-ATGCAGCTAATACGACTCACTATAGGCAGATGATGTCCTTA-3ʹ

**U35a_R –** 5ʹ-CTCCTGGCATCAGCTAAGCCATTGGCATTGTCGCCATTCCCACAG-3ʹ

***U12 snRNA***

**U12_T7**– 5ʹ-ATGCAGCTAATACGACTCACTATAGGGTGCCTTAAACTTATGAG-3ʹ

**U12_R** – 5ʹ-CGGGCAGATCGCAACTCCCAGGCATCCCGCAAAG-3ʹ
